# Solitary meat-eaters: solitary, carnivorous carnivorans exhibit the highest degree of sexual size dimorphism

**DOI:** 10.1038/s41598-019-51943-x

**Published:** 2019-10-25

**Authors:** Chris J. Law

**Affiliations:** Department of Mammalogy and Division of Paleontology, American Museum of Natural History, 200 Central Park West, New York, NY 10024 USA

**Keywords:** Evolution, Zoology

## Abstract

Although sexual size dimorphism (SSD) is widespread across the animal tree of life, the underlying evolutionary processes that influence this phenomenon remains elusive and difficult to tease apart. In this study, I examined how social system (as a proxy for sexual selection) and diet (as a proxy for natural selection) influenced the evolution of SSD in terrestrial carnivorans (Carnivora; Mammalia). Using phylogenetic comparative methods, I found that are territorial solitary and carnivorous carnivorans exhibited selection towards increased degree of male-biased SSD compared to other carnivorans with alternative social systems and diets. I also found the absence of Rensch’s rule across most carnivoran clades, suggestion a relaxation of the influences of sexual selection on SSD. These results together suggest that sexual selection and niche divergence together are important processes influencing the evolution of male-biased SSD in extant terrestrial carnivorans.

## Introduction

Although sexual size dimorphism (SSD) is widespread across the animal tree of life^1^, the underlying evolutionary processes that influence this phenomenon remains elusive and difficult to tease apart. Sexual selection and natural selection are often viewed as the primary mechanisms underlying SSD by influencing body size in females, males, or both across evolutionary time. In most endothermic vertebrates (i.e. mammals and birds), sexual selection has long been hypothesized to be the primary driver of SSD^2,3^, in which the larger sex increases their reproductive success directly through intrasexual competition^4–8^. Sexual selection can also lead to the reduction of body size in a sex if a smaller size enhances courtship displays^9^. Natural selection through fecundity selection and/or intersexual niche divergence has also been hypothesized to influence or maintain the evolution of SSD. Under fecundity selection, female-biased size dimorphism is hypothesized to increase reproductive rates in larger females^2,10^. In contrast, niche divergence is hypothesized to reduce intersexual competition for resources through ecological niche divergence between the sexes^11–14^; however, evidence for this hypothesis has been fewer.

The majority of previous work have supported the sexual selection hypothesis by finding a relationship between greater degree of SSD and increased polygynous mating systems^4,5,8,15^. Further evidence for sexual selection is also found in Rensch’s rule, which states that SSD increases with body size in species with male-biased dimorphism (hyperallometry) but decreases with body size in species with female-biased dimorphism (hypoallometry)^16,17^. Because larger species tend to be more polygynous than smaller species, strong sexual selection is expected to influence greater degrees of SSD in larger species^4,9,15,18^. Therefore, hyperallometric patterns in both mammals and birds also suggest that sexual selection influences the evolution of SSD^4,8^. However, researchers have also found inconsistent relationships between the degree of SSD and the degree of polygynous mating systems; i.e. the most polygynous or territorial species are not always the most sexually dimorphic and monogamous species are not always the least sexually dimorphic^4,14,19,20^. These inconsistent patterns suggest that additional evolutionary processes may also influence the evolution and maintenance of SSD. Similarly, not all clades follow Rensch’s rule^21–24^; that is, larger species do not always exhibit greater degree of SSD as expected by sexual size allometry. This suggests a relaxation of sexual selection in larger species or an increase in SSD in smaller species through other mechanisms such as niche divergence.

Within carnivoran mammals (Carnivora), male-biased SSD is often attributed to sexual selection where males compete for mating success and larger male body sizes are selected for^5,19,25^. Pinnipeds (seals, sea lions, and the walrus) are the textbook example of the sexual selection hypothesis, as they exhibit a strong relationship between the degree of SSD and polygyny^5,7,26^. In terrestrial carnivorans, researchers similarly hypothesized that the greatest degree of male-biased SSD should occur in species that are highly territorial and exhibit polygynous mating systems. However, the pattern between social/mating system and the degree of sexual dimorphism is not consistent among terrestrial carnivoran clades^4,14,20,22,27^. Furthermore, Carnivora does not follow Rensch’s rule^4^, suggesting that alternative processes may also influence SSD. In the carnivoran clades Musteloidea and Canidae, the evolution and maintenance of sexual dimorphism appears to be influenced by the degree of carnivory^14,22,27^. This pattern is attributed to the hypothesis that competition for terrestrial vertebrate prey is greater than plant material and non-vertebrate prey^23,28,29^. Therefore, under the niche divergence hypothesis, increased sexual dimorphism in carnivorous carnivorans is assumed to reduce intraspecific dietary competition by reducing competition between the sexes. Supporting this assumption are observations finding that females and males of many carnivoran species utilize different dietary resources^30–33^.

Whether sexual selection and/or niche divergence best predicts SSD across the entire carnivoran clade remains to be investigated. Here, I examined how social systems (a proxy for sexual selection) and diet (a proxy for niche divergence) influenced the evolution of SSD in terrestrial carnivoran mammals. I used phylogenetic comparative methods to test two hypotheses: (1) SSD scales allometrically with respect to species body size both within carnivoran families and (2) social system and/or diet influenced the evolution of SSD. To test hypothesis 1, I used reduced major axis regressions to examine the relationship between female and male body masses across each clade. To test hypothesis 2, I used Ornstein-Uhlenbeck modeling approaches to examine how social systems and diet correspond with the evolution of SSD across Carnivora. If sexual selection is the primary force influencing SSD, I predict that the degree of SSD should be greatest in territorial solitary carnivorans and lowest in pair-living carnivorans, suggesting that SSD may have evolved as a response to male–male competition. If niche divergence is the primary force influencing SSD, I predict that the degree of SSD, either male-biased of female-biased, should be greatest in terrestrial carnivorous carnivorans whereas the SSD should be lowest in carnivorans that feed on nonvertebrate prey and plant material that more abundant and easier to obtain. This would suggest that intraspecific competition for less abundant resources facilitates the evolution of SSD.

## Methods

Data on female and male body masses, diet, and social systems were obtained from the literature, primarily from the *Handbook of the Mammals of the World*^34^, Johnson *et al*.^22^, and Noonan *et al*.^27^ (see Supplementary Table S1 for all references). I was able to obtain all these data from 166 carnivorans (~60% of carnivoran diversity) stemming from all terrestrial carnivoran families with the exception of Nandiniidae and Prionodontidae, both of which are single genera clades. I did not include pinnipeds (seals, sea lions, and the walrus) because this clade of fully aquatic carnivorans exhibits several apomorphic behaviors, morphologies, and ecologies adapted for aquatic habitats. Furthermore, there is already strong evidence of a link between the degree of sexual dimorphism and harem size, suggesting that sexual selection is the primary mechanism influencing and/or maintaining SSD in pinnipeds^5,7,26^. Because the majority of terrestrial carnivorans do not exhibit harem behavior, attempting to combine the two groups of carnivorans with very different mating/social systems would lead to conflicting results with respect to the relationship between SSD and mating/social systems.

I assessed the degree of SSD using the size dimorphism index (SDI)^35^:

$$SDI=\pm \,(\frac{{S}_{L}}{{S}_{S}}-1)\ast 100,$$

where S_L_ and S_S_ are the mean body mass of the larger and smaller sex, respectively. A positive sign is assigned if the male trait is larger whereas a negative sign is assigned if the female trait is larger. There is no sexual dimorphism if SDI = 0%.

Mating systems is an ideal proxy for sexual selection. Unfortunately, the degree of polygyny for the majority of carnivorans is unknown. Therefore, I used social system as a proxy of sexual selection^22,27^. Carnivorans have varied social systems ranging from highly solitary species that exhibit intrasexual territoriality in which one male defends a territory that may contain multiple female territories to highly social species that tend to be monogamous or promiscuous^34^. Following previous work examining carnivoran sexual dimorphism^22,27^, I used Ortolani and Caro’s^36^ categorical scheme to categorize the 166 carnivorans into one of four social systems: territorial solitary where species exhibit predominantly solitary life-histories with defended territories, pair-living where species exhibit predominantly monogamous mating life-histories, group-living where species exhibit predominantly obligate groups with members exhibiting monogamous mating behaviors and/or family groups, and variable groups where species exhibit both solitary and group-living life-histories that can be variable across populations. Although lions (*Panthera leo*) live in prides, I classified the species as territorial solitary because male lions defend the pride’s territory against other male competitors^34^.

I used Gittleman^37^ and Van Valkenburgh’s^38^ categorical schemes to categorize the 166 carnivorans into one of five dietary regimes: terrestrial carnivory (hypercarnivory) with diets consisting of >70% terrestrial vertebrates, omnivory (mesocarnivory) with diets consisting of 50–70% terrestrial vertebrates, insectivory with diets consisting of >60% arthropods, herbivory with diets consisting of >90% plant material, and aquatic carnivory with diets consisting of >90% aquatic prey. I also categorized the 166 carnivorans into regimes that consisted of the interaction between social system and diet. However, these interactions created 18 different regimes, many of which contained less than four species. Because 18 regimes were far too many for current comparative methods, I reduced the number of regimes by aggregating pair-living and variable groups with group-living and by combining insectivory, aquatic carnivory, and herbivory into a new “other” category. This resulted in a new categorical scheme that contained six regimes: group-living carnivory, group-living omnivory, group-living other, solitary carnivory, solitary omnivory, and solitary other.

All subsequent analyses were performed under a phylogenetic framework using the most recent time-calibrated phylogeny of terrestrial carnivorans^39^.

### Hypothesis 1: SSD within carnivoran families scales allometrically with respect to species body size (Rensch’s rule)

Following the recommendations of Abouheif and Fairbairn^17^, I examined predictions of Rensch’s rule by using phylogenetic reduced major axis (RMA) regressions to regress ln female body mass on ln male body mass and test if the slope deviated from the null hypothesis of a slope = 1. A slope significantly greater than 1 would confirm the presence of Rensch’s rule where SSD increases with increasing body size across carnivorans. I performed the RMA regression on the entire carnivoran clade and within each carnivoran family. All regression parameters were simultaneously estimated with phylogenetic signal in the residual error as Pagel’s^40^ lambda^41^ using the R package phytools^42^.

### Hypothesis 2: Social system and/or diet influenced the evolution of SSD

I tested the hypothesis that social system and/or diet influenced the evolution of SSD in carnivorans by using generalized evolutionary modeling^43,44^. I first generated two sets of 500 stochastically mapped trees of the social system and dietary regimes with the make.simmap function^45^ in phytools^42^. Each set of 500 trees was then summarized to estimate the ancestral states of social system and dietary regimes. I then fit five evolutionary models to my SDI dataset: (1) single-rate Brownian (BM1) model that assumes SSD variance accumulates proportional to evolutionary time under a random walk, (2) a single-optimum Ornstein–Uhlenbeck (OU1) model that constrains SSD to evolve toward one optimum (θ) across the entire carnivoran phylogeny, (3) a multi-optima OU model that allows species in each social system regime (OUM_social system_) to exhibit different independent SSD optima, (4) a multi-optima OU model that allows species in each dietary regime (OUM_diet_) to exhibit different independent SSD optima, and (5) a multi-optima OU model that allows species in each social system x dietary regime (OUM_social_diet_) to exhibit different independent SSD optima. All model fitting were performed with the R package OUwie^46^. I fit all models across 500 stochastically mapped trees to take into account uncertainty in the ancestral character states, and I evaluated the best-fitting model using corrected Aikaike Information Criterion weights (AICcW). Support for the BM1 or OU1 models would suggest that SSD evolved independently of social system or dietary regimes whereas support for the OUM_social system_, OUM_diet_, or OUM_social_diet_ models would suggest that social system and/or dietary regimes influenced the evolution of SSD. Lastly, I generated a 95% confidence interval for all model parameters (Θ, α, and σ^2^) of the best-fit model by performing parametric bootstrapping for 1000 replicates using the function OUwie.boot.

Additional factors aside from sexual selection and niche divergence may also influence the evolution of SSD. These hidden processes may not be captured by my *a priori* hypotheses that social system and/or dietary regimes influenced SSD evolution and may therefore unintentionally hide patterns of phylogenetic natural history that are biologically relevant^47^. Therefore, I also used bayou^48^ v2.1.1 to examine evolutionary shifts in SSD. Bayou uses a reversible-jump MCMC to fit multi-peak OU models to estimate the placement and magnitude of regime shifts without *a priori* groupings of social system and/or dietary ecology. I placed a Poisson prior with lambda = 15 on the number of shifts between adaptive regimes and allowed only one shift per branch with equal probability that each branch has a shift. I ran two independent MCMC chains with one million generations each sampled every 10,000 and examined if the two chains converged using Gelman and Rubin’s R statistic. All effective sample sizes were >200 after discarding the first 30% of samples as burn-in. I reported only evolutionary shifts with a posterior probability (pp) > 0.5.

## Results

### Hypothesis 1: SSD within terrestrial carnivoran families scales allometrically with respect to species body size (Rensch’s rule)

**Figure 1:**
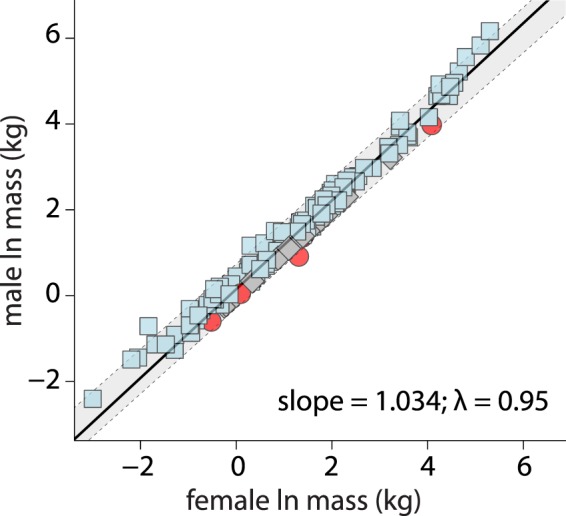
Scaling of female body mass on male body mass across carnivorans using phylogenetic reduced major axis (RMA) regression. Slope was not significantly different from 1 (0.056).

The RMA regression slope between ln female mass and ln male mass across terrestrial carnivorans was not significantly different from 1.00 (slope [±standard error] = 1.03 [±0.01], T = 1.93, df = 105.62, P = 0.056; Fig. 1), suggesting that carnivorans do not follow predictions of Rensch’s rule of increased male-biased SSD with increasing body size. Examination of each carnivoran family found different Rensch trends: Felidae (slope = 1.10 [±0.03], P = 0.004) and Ursidae (slope = 1.56 [±0.11], P = 0.001) exhibited a positive Rensch trends, Viverridae (slope = 0.83 [±0.06], P = 0.037) exhibited a negative Rensch trend, but the remaining families exhibited no significant differences with respect to isometry (Table 1). A potential caveat to interpretations of these regressions is that seven of these families exhibited fewer than nine species.

**Table 1:** Estimated slopes from phylogenetic reduced major axis (RMA) regressions to determine if Rensch’s rule is present across Carnivora and within carnivoran families.

Clade	# of species	slope ± SE	λ	R^2^	T	df	P	Rensch’s Rule
Carnivora	166	1.03 ± 0.1	0.95	0.95	1.93	105.6	0.056	No
Ailuridae	1							n/a
Canidae	35	1.03 ± 0.3	0.62	0.98	1.27	22.14	0.218	No
Eupleridae	6	1.11 ± 0.06	0.97	0.99	1.81	4.006	0.145	No
Felidae	33	1.10 ± 0.03	0.83	0.97	3.16	22.19	**0**.**004**	Positive
Herpestidae	14	0.97 ± 0.08	0.00	0.92	0.4	9.523	0.697	No
Hyaenidae	3	0.96 ± 0.11	0.00	0.99	0.32	2.669	0.773	No
Mephitidae	7	0.93 ± 0.5	0.00	0.99	1.49	4.005	0.211	No
Mustelidae	43	0.98 ± 0.04	0.84	0.94	0.49	29.19	0.626	No
Procyonidae	7	1.10 ± 0.12	0.00	0.95	0.97	4.707	0.381	No
Ursidae	8	1.56 ± 0.11	1.00	0.97	6.38	6.039	**0**.**001**	Positive
Viverridae	9	0.83 ± 0.06	0.73	0.97	2.66	6.038	**0**.**037**	Negative

P-values reflect differences from the null hypothesis where the slope = 1. Bold P values indicate significance (α = 0.05).

### Hypothesis 2: Social system and/or diet influenced the evolution of SSD

**Figure 2:**
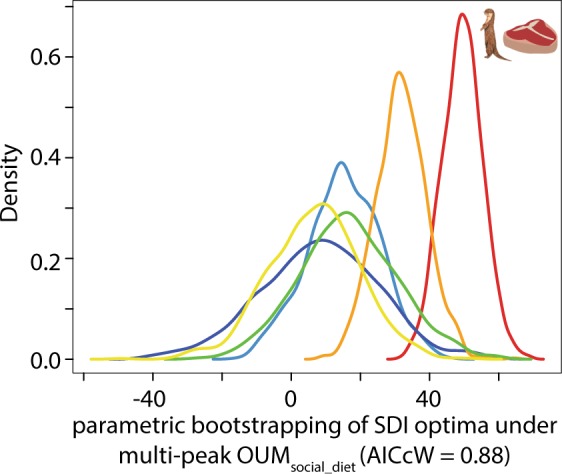
Density plot of the parametric bootstrap of estimated optima from the best-fitting generalized Ornstein–Uhlenbeck (OU) model of sexual size dimorphism. A multi-peak OU model with separate optima between carnivorans that are both solitary and carnivorous (red) and carnivorans with alternative social systems and diets was the best-fitting model (AICcW = 0.88). Orange = group-living and carnivorous; blue = group-living and omnivorous; purple = solitary and omnivorous; green = group-living and other; yellow = solitary and other.

The best supported model was the multi-peak OUM_social_diet_ model (AICcW = 0.88; Table 2) with a phylogenetic half-life (ln(2)/α) of 3.16 Myr. This model with parametric bootstrapping revealed that solitary carnivorous carnivorans exhibited the greatest degree of SSD (Θ_sol-car_ = 49.89% [38.10%, 60.84%]) followed by solitary omnivorous carnivorans (Θ_sol-omn_ = 32.61% [17.48%, 46.65%]). In contrast, parametric bootstrapping indicated that the optimal SSD for the remaining regimes were largely overlapping; nevertheless, all exhibited lower degrees of male-biased SSD (Θ_sol-oth_ = 6.27% [−19.88%, 32.31%], Θ_grp-car_ = 15.04% [−6.15%, 35.90%], Θ_grp-omn_ = 8.93% [−25.83%, 40.62%], and Θ_grp-oth_ = 16.86% [−11.03%, 45.73%]; Fig. 2).

**Table 2:** Comparisons of evolutionary model fits for sexual size dimorphism (SSD).

Model	AICc	ΔAICc	AICcW
BM1	1524.76	47.14	0.00
OU1	1484.70	7.08	0.04
OUM_group_	1484.87	7.25	0.04
OUM_diet_	1484.93	7.31	0.04
OUM_social_diet_	1477.62	0.00	0.88

Mean Akaike information criterion (AICc) were calculated from the 500 replications. ΔAICc is the model’s mean AICc minus the minimum AICc between models.

**Figure 3:**
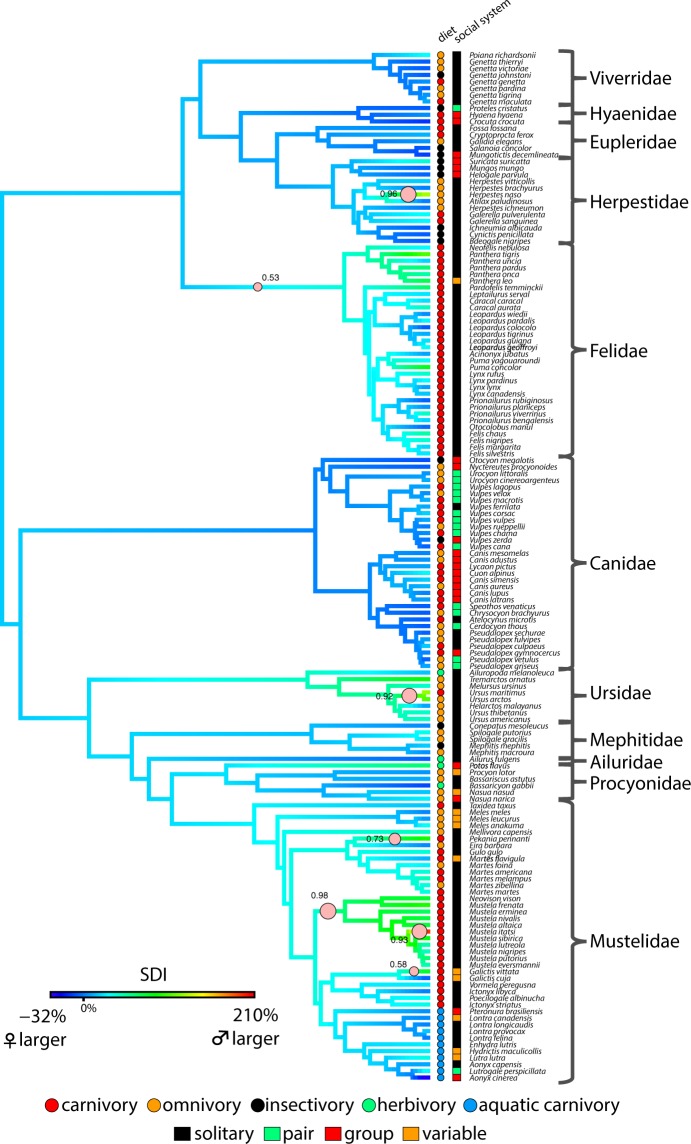
Seven transitions toward sexual size dimorphism (SSD) increases (pink circles) occurred across the pruned carnivoran phylogeny. Circle sizes represent posterior probability (all pp > 0.5). Branch colors represent the ancestral state reconstruction of SSD via the size dimorphic index (SDI).

The data-driven approach of bayou revealed seven independent shifts towards increased male-biased SSD (all pp > 0.5; Fig. 3). The family Felidae and the subfamily Mustelinae (within Mustelidae) were the only carnivoran clades to exhibit increased SSD. The remaining shifts occurred in branches towards increased SSD in single species or species pairs within Mustelidae, Ursidae, and Herpestidae. All species with evolutionary shifts towards increased SSD were solitary and/or carnivorous (Fig. 3).

**Figure 4:**
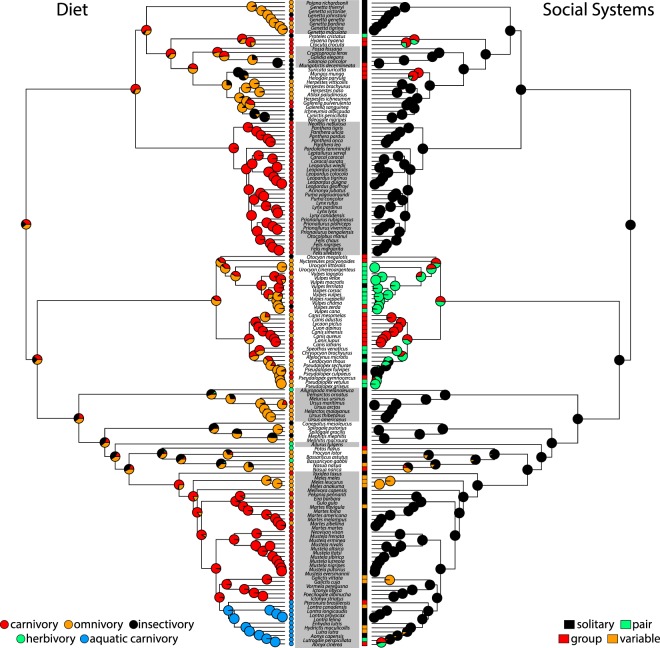
Ancestral state reconstruction of dietary and social system regimes mapped onto the pruned carnivoran phylogeny. Pie charts on each node show the relative Bayesian posterior probability of each character state across 500 simulations.

The ancestral state reconstruction of social systems across 500 simulations suggest that the solitary condition was the dominant and most ancestral state in Carnivora (Fig. 4). Pair, group, and variable systems evolved from the solitary condition an average of 5.6, 3.6, and 8.3 times, respectively. Transitions back to solitary states were rare. Pair and group social systems primarily occurred within Canidae. In contrast, there was uncertainty in the ancestral state of diet, with nearly equal probability that carnivory, omnivory, or insectivory was the ancestral state (Fig. 4).

## Discussion

### Evolution of carnivoran sexual dimorphism

Sexual selection is often viewed as the major driver of the evolution of sexual dimorphism^2,3^. However, an increasing number of studies have begun to recognize niche divergence as an important mechanism that also contributes to the evolution and maintenance of SSD^11–14^. In this study, I found that social systems and diet—proxies for sexual selection and niche divergence, respectively—influenced the evolution and maintenance of SSD across Carnivora. My OU modeling indicated that carnivorans that were terrestrial solitary and carnivorous exhibited selection towards increased degree of SSD compared to other carnivorans with alternative social systems and diets. The phylogenetic half-life for this model was relatively short (3.16 Myr), indicating that there was fast selection towards increased male-biased SSD across extant carnivorans. The data-driven approach with bayou confirms this finding as five of the seven shifts to increased SSD occurred on branches of carnivorans that are both solitary and carnivorous. Furthermore, these five shifts contain 45 of the 65 carnivorans that were both solitary and carnivorous in this study. The remaining two shifts lead to clades/species that were either solitary or carnivorous. The absence of Rensch’s rule in the majority of the carnivoran families are consistent with these results; because the degree of carnivory is not a function a carnivoran size^49,50^, increased SSD due to niche divergence can occur in small carnivorous species and erase the trend that is predicted by Rensch’s rule.

My results indicate that both sexual selection and niche divergence simultaneously influence increases in male-biased SSD over evolutionary time. However, ancestral state reconstruction also lend some support to the postulation that SSD first evolved as a response to sexual selection, and subsequent intersexual differences secondarily reinforced or maintained SSD by allowing females and males to exploit different resources (i.e. niche divergence)^13,14,20,51^. Solitary, territorial systems is the ancestral condition in Carnivora, whereas terrestrial carnivory within extant carnivoran clades did not evolve until the Late Oligocene and Early Miocene (Fig. 4). Therefore, it is tempting to speculate that sexual selection was the original mechanism that drove increased male-biased SSD in territorial solitary carnivorans where males aggressively defend territories against male conspecifics^13,25^. In turn, the distribution of female and male territories may have also helped maintain SSD through niche divergence. Because male territories are larger and overlap with multiple smaller female territories^52–54^, male conspecifics exhibit larger range sizes and potentially encounter a wider range of prey. As a result, male conspecifics can exploit these novel, often larger prey items^30–33,55,56^ due to morphological and functional adaptations (e.g. larger bite forces, stronger skulls and teeth) associated with their larger body sizes^13,14,57–59^. Under the niche divergence hypothesis, partitioning of prey items between males and females then further maintained SSD by reducing dietary competition between the female and male conspecifics. Niche divergence is hypothesized to act more strongly on carnivorous species because competition for vertebrate prey is greater than plant material and non-vertebrate prey^23,28,29^. Unsurprisingly, carnivory appears to have evolved independently multiple times across Carnivora rather than as a single origin, and these shifts occur primarily within carnivorans with solitary, terrestrial social systems (Fig. 4). However, the incorporation of the fossil record, specifically information on the social behaviors, dietary ecologies, and SSD in extinct carnivorans, are needed to truly test these patterns.

Felids and musteline weasels (*Mustela* sp.) are the two largest clades that display increased degrees of SSD (Fig. 3), and their natural histories fit the “sexual selection then niche divergence” framework. Species of both clades are largely polygynous, and under sexual selection, larger males are more successful in defending territories against other males^60–62^. With larger territories, male conspecifics encounter and exploit different, often larger prey^30–33,63^ by presumably using their larger bodies, skulls, teeth, and biting abilities to capture and consume these different prey^14,57–59^. Because all felids and musteline weasels are hypercarnivorous and specialize on vertebrate prey, niche divergence is hypothesized to reduce intraspecific competition for food by further maintaining size dimorphism in these traits^14,57,58^. Similar patterns are found in other solitary, carnivorous carnivorans that display increased SSD (Fig. 3). For example, male polar bears tend to eat larger 400-kg bearded seals, whereas females prefer smaller 60 kg ringed seals^56^.

### Influences of fecundity selection?

The majority of sexual dimorphism studies in mammals examine the mechanisms that select for larger male body sizes. However, fecundity selection acting on female body size may also influence the evolution and maintenance of SSD. Unlike most animals^64–67^, fecundity in mammals decreases with increasing female body size between species^4,68,69^; therefore, fecundity selection is hypothesized to reduce female body size in mammals^4^. Contrary to expectations, fecundity does not decrease with increasing female body mass across the entire carnivoran clade^4^. However, these trends differ between the carnivoran families. Mustelids exhibit a negative trend between litter size (proxy for fecundity) and female body size, suggesting that small female body sizes are favored^27^. Previous researchers support this hypothesis, positing that small female conspecifics are selected to reduce the high energetic demands associated with reproduction and rearing of young^51,63,70,71^. Most mustelids exhibit elongate body plans^72^ and therefore increased energy requirements imposed by an increased surface-to-volume ratio^73–75^. Rearing of young further increases these energetic demands; for example, lactating southern sea otters (*Enhydra lutris neresis*) exhibit daily energy demands 85–110% higher than non-reproductive females^76,77^. Therefore, selection for smaller female body sizes would theoretically reduce the absolute food requirements needed to rear young^51,63,70,71^. In contrast, felids do not exhibit a significant trend between litter size and female body size^22^ whereas canids exhibits a positive trend between litter size and female body size^22,78^. These results suggest that fecundity selection may not be a strong mechanism in maintaining carnivoran SSD or that it affects SSD on a clade-by-clade basis. Nevertheless, examinations of the influence of fecundity selection on mammalian sexual dimorphism are rare relative to studies on sexual selection and niche divergence, and additional natural history and quantification of fecundity are needed to fully test the influences of fecundity selection on sexual dimorphism.

### Conclusion, caveats, and future directions

In this study, I found evidence that both sexual selection and niche divergence drove and maintained SSD in extant carnivorans, where sexual selection may have first drove increased male-biased SSD in territorial solitary carnivorans, and subsequent selection from niche divergence further reinforced or increased SSD in carnivorous carnivorans. Nevertheless, the interaction of sexual selection and niche divergence and its influence on the evolution of SSD requires continual investigation, as both processes can work simultaneously to reinforce or increase SSD across evolutionary time^79^. Two caveats to this study are 1) the use of social system as a proxy of sexual selection rather than mating system and 2) the assumption that there is ecological significance in dietary partitioning between males and females. Unfortunately, information on both mating systems and intersexual niche divergence are largely unknown in most carnivorans. A third caveat to this study and nearly all other studies of sexual dimorphism evolution is the absence of fossil data. Previous work demonstrated that incorporating the fossil record in macroevolutionary analyses dramatically improves model selection of trait evolution and ancestral state reconstructions^80–82^. Within studies pertaining to sexual dimorphism, the fossil record has primarily been used to understand the evolution of sexual dimorphism in primates and hominids^83–87^. Nevertheless, sexual dimorphism has been described in an increasing number of extinct carnivorans^58,88–90^ and can be used to understand the evolution of sexual dimorphism under a phylogenetic framework (e.g. Cullen *et al*.^26^). The continual development of new comparative methods combining paleontological and neontological data will allow researchers to test more robust and informed hypotheses pertaining to the evolution and maintenance of sexual dimorphism.

## Supplementary information

Supplementary Information
