# Supplementary material for: Solitary meat-eaters: solitary, carnivorous carnivorans exhibit the highest degree of sexual size dimorphism
Source: Sci Rep. 2019 Oct 25;9:15344. doi: 10.1038/s41598-019-51943-x (PMC6814822; doi:10.1038/s41598-019-51943-x)
Supplement: Supplementary file 1 — Supplementary Information [file 41598_2019_51943_MOESM1_ESM.pdf]

Supplementary Info

Solitary meat-eaters: solitary, carnivorous carnivorans exhibit the highest degree of sexual size dimorphism

Running title: Sexual dimorphism evolution in Carnivora

Chris Law*

American Museum of Natural History, 200 Central Park West, New York, NY 20024

*claw@amnh.org

Figure S1. Scaling of female body mass on male body mass across carnivoran familes using phylogenetic reduced major axis (RMA) regression.

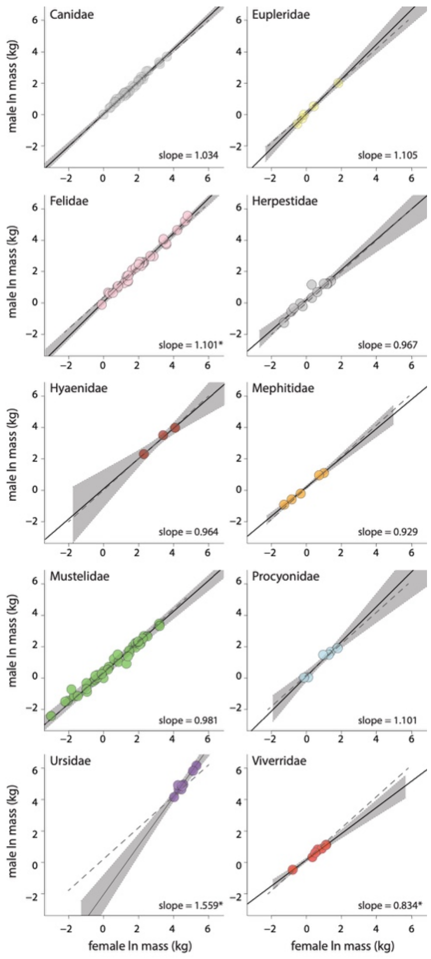

Table S1. Body masses, dietary regimes, and social system for each species.

Species	Family	♀ mass	♂ mass	SDI	Diet	Social System	References
Ailurus_fulgens	Ailuridae	4.9	5	2.041	Herbivory	Solitary	(Wilson *et al.*, 2009; Noonan *et al.*, 2016)
Atelocynus_microtis	Canidae	9.5	9.5	0	Carnivory	Solitary	(Wilson *et al.*, 2009; Johnson *et al.*, 2017)
Canis_adustus	Canidae	8.3	9.4	13.253	Omnivory	Group	(Wilson *et al.*, 2009; Johnson *et al.*, 2017)
Canis_aureus	Canidae	7.3	8.8	20.548	Omnivory	Group	(Wilson *et al.*, 2009; Johnson *et al.*, 2017)
Canis_latrans	Canidae	10.1	11.6	14.851	Carnivory	Group	(Wilson *et al.*, 2009; Johnson *et al.*, 2017)
Canis_lupus	Canidae	37	40	8.108	Carnivory	Group	(Wilson *et al.*, 2009; Johnson *et al.*, 2017)
Canis_mesomelas	Canidae	7.4	8.1	9.459	Omnivory	Group	(Wilson *et al.*, 2009; Johnson *et al.*, 2017)
Canis_rufus	Canidae	24.3	28.5	17.284	Carnivory	Group	(Wilson *et al.*, 2009; Johnson *et al.*, 2017)
Canis_simensis	Canidae	12.8	16.2	26.563	Carnivory	Group	(Wilson *et al.*, 2009; Johnson *et al.*, 2017)
Cerdocyon_thous	Canidae	5.7	5.7	0	Omnivory	Pair	(Wilson *et al.*, 2009; Johnson *et al.*, 2017)
Chrysocyon_brachyurus	Canidae	25	25	0	Omnivory	Pair	(Wilson *et al.*, 2009; Johnson *et al.*, 2017)
Cuon_alpinus	Canidae	11.5	15.8	37.391	Carnivory	Group	(Wilson *et al.*, 2009; Johnson *et al.*, 2017)
Lycaon_pictus	Canidae	24	28	16.667	Carnivory	Group	(Wilson *et al.*, 2009; Johnson *et al.*, 2017)
Nyctereutes_procyonoides	Canidae	4.5	4.5	0	Omnivory	Group	(Wilson *et al.*, 2009; Johnson *et al.*, 2017)

Otocyon_megalotis	Canidae	4.1	4	-2.439	Insectivory	Group	(Wilson *et al.*, 2009; Johnson *et al.*, 2017)
Pseudalopex_culpaeus	Canidae	8.5	11	29.412	Carnivory	Solitary	(Wilson *et al.*, 2009; Johnson *et al.*, 2017)
Pseudalopex_fulvipes	Canidae	2.9	3.3	13.793	Omnivory	Solitary	(Wilson *et al.*, 2009; Johnson *et al.*, 2017)
Pseudalopex_griseus	Canidae	3.3	4	21.212	Omnivory	Pair	(Wilson *et al.*, 2009; Johnson *et al.*, 2017)
Pseudalopex_gymnocercus	Canidae	4.2	4.6	9.524	Carnivory	Group	(Wilson *et al.*, 2009; Johnson *et al.*, 2017)
Pseudalopex_sechurae	Canidae	3.6	3.6	0	Omnivory	Solitary	(Wilson *et al.*, 2009; Johnson *et al.*, 2017)
Pseudalopex_vetulus	Canidae	3.4	3.3	-2.941	Omnivory	Pair	(Wilson *et al.*, 2009; Johnson *et al.*, 2017)
Speothos_venaticus	Canidae	6.5	6.5	0	Carnivory	Pair	(Wilson *et al.*, 2009; Johnson *et al.*, 2017)
Urocyon_cinereoargenteus	Canidae	3.3	4	21.212	Omnivory	Pair	(Wilson *et al.*, 2009; Johnson *et al.*, 2017)
Urocyon_littoralis	Canidae	1.8	2	11.111	Omnivory	Pair	(Wilson *et al.*, 2009; Johnson *et al.*, 2017)
Vulpes_bengalensis	Canidae	2	2.95	47.5	Omnivory	Pair	(Wilson *et al.*, 2009; Johnson *et al.*, 2017)
Vulpes_cana	Canidae	1	1	0	Omnivory	Pair	(Wilson *et al.*, 2009; Johnson *et al.*, 2017)
Vulpes_chama	Canidae	2.5	2.8	12	Omnivory	Pair	(Wilson *et al.*, 2009; Johnson *et al.*, 2017)
Vulpes_corsac	Canidae	2.1	2.7	28.571	Carnivory	Pair	(Wilson *et al.*, 2009; Johnson *et al.*, 2017)
Vulpes_ferrilata	Canidae	3.5	4.1	17.143	Carnivory	Solitary	(Wilson *et al.*, 2009; Johnson *et al.*, 2017)
Vulpes_lagopus	Canidae	3.1	3.6	16.129	Carnivory	Pair	(Wilson *et al.*, 2009; Johnson *et al.*, 2017)

Vulpes_macrotis	Canidae	1.9	2.3	21.053	Carnivory	Pair	(Wilson *et al.*, 2009; Johnson *et al.*, 2017)
Vulpes_pallida	Canidae	2.8	2.8	0	Omnivory	Pair	(Wilson *et al.*, 2009; Johnson *et al.*, 2017)
Vulpes_rueppellii	Canidae	1.5	1.6	6.667	Omnivory	Pair	(Wilson *et al.*, 2009; Johnson *et al.*, 2017)
Vulpes_velox	Canidae	2	2.2	10	Omnivory	Pair	(Wilson *et al.*, 2009; Johnson *et al.*, 2017)
Vulpes_vulpes	Canidae	5.3	6.3	18.868	Carnivory	Pair	(Wilson *et al.*, 2009; Johnson *et al.*, 2017)
Vulpes_zerda	Canidae	1.4	1.5	7.143	Insectivory	Group	(Wilson *et al.*, 2009; Johnson *et al.*, 2017)
Cryptoprocta_ferox	Eupleridae	6.15	7.4	20.325	Carnivory	Solitary	(Wilson et al., 2009)
Fossa_fossana	Eupleridae	1.525	1.7	11.475	Carnivory	Solitary	(Wilson et al., 2009)
Galidia_elegans	Eupleridae	0.87	0.992	14.023	Omnivory	Solitary	(Wilson et al., 2009)
Galidictis_grandidieri	Eupleridae	1.494	1.723	15.328	Insectivory	Solitary	(Wilson et al., 2009)
Mungotictis_decemlineata	Eupleridae	0.595	0.55	-7.563	Insectivory	Group	(Wilson et al., 2009)
Salanoia_concolor	Eupleridae	0.78	0.78	0	Insectivory	Solitary	(Wilson et al., 2009)
Acinonyx_jubatus	Felidae	35.9	41.4	15.32	Carnivory	Solitary	(Wilson *et al.*, 2009; Johnson *et al.*, 2017)
Caracal_aurata	Felidae	7.2	11	52.778	Carnivory	Solitary	(Wilson *et al.*, 2009; Johnson *et al.*, 2017)
Caracal_caracal	Felidae	10	12.9	29	Carnivory	Solitary	(Wilson *et al.*, 2009; Johnson *et al.*, 2017)
Felis_chaus	Felidae	5.1	8.1	58.824	Carnivory	Solitary	(Wilson *et al.*, 2009; Johnson *et al.*, 2017)

Felis_margarita	Felidae	2.2	2.78	26.364	Carnivory	Solitary	(Wilson *et al.*, 2009; Johnson *et al.*, 2017)
Felis_nigripes	Felidae	1.3	1.93	48.462	Carnivory	Solitary	(Wilson *et al.*, 2009; Johnson *et al.*, 2017)
Felis_silvestris	Felidae	3.7	4.9	32.432	Carnivory	Solitary	(Wilson *et al.*, 2009; Johnson *et al.*, 2017)
Leopardus_colocolo	Felidae	4	3.9	-2.5	Carnivory	Solitary	(Wilson *et al.*, 2009; Johnson *et al.*, 2017)
Leopardus_geoffroyi	Felidae	3.8	5.5	44.737	Carnivory	Solitary	(Wilson *et al.*, 2009; Johnson *et al.*, 2017)
Leopardus_guigna	Felidae	1.4	1.8	28.571	Carnivory	Solitary	(Wilson *et al.*, 2009; Johnson *et al.*, 2017)
Leopardus_pardalis	Felidae	9.8	13.5	37.755	Carnivory	Solitary	(Wilson *et al.*, 2009; Johnson *et al.*, 2017)
Leopardus_tigrinus	Felidae	2.2	2.6	18.182	Carnivory	Solitary	(Wilson *et al.*, 2009; Johnson *et al.*, 2017)
Leopardus_wiedii	Felidae	3.3	3.6	9.091	Carnivory	Solitary	(Wilson *et al.*, 2009; Johnson *et al.*, 2017)
Leptailurus_serval	Felidae	8.5	11.2	31.765	Carnivory	Solitary	(Wilson *et al.*, 2009; Johnson *et al.*, 2017)
Lynx_canadensis	Felidae	8.6	10.7	24.419	Carnivory	Solitary	(Wilson *et al.*, 2009; Johnson *et al.*, 2017)
Lynx_lynx	Felidae	17.3	19.6	13.295	Carnivory	Solitary	(Wilson *et al.*, 2009; Johnson *et al.*, 2017)
Lynx_pardinus	Felidae	9.3	12.8	37.634	Carnivory	Solitary	(Wilson *et al.*, 2009; Johnson *et al.*, 2017)
Lynx_rufus	Felidae	6.2	9.3	50	Carnivory	Solitary	(Wilson *et al.*, 2009; Johnson *et al.*, 2017)
Neofelis_diardi	Felidae	16.8	23	36.905	Carnivory	Solitary	(Wilson *et al.*, 2009; Johnson *et al.*, 2017)
Neofelis_nebulosa	Felidae	14.4	19.6	36.111	Carnivory	Solitary	(Wilson *et al.*, 2009; Johnson *et al.*, 2017)

Otocolobus_manul	Felidae	4.02	4.12	2.488	Carnivory	Solitary	(Wilson *et al.*, 2009; Johnson *et al.*, 2017)
Panthera_leo	Felidae	104.6	185.6	77.438	Carnivory	Solitary	(Wilson *et al.*, 2009; Johnson *et al.*, 2017)
Panthera_onca	Felidae	66.9	104.5	56.203	Carnivory	Solitary	(Wilson *et al.*, 2009; Johnson *et al.*, 2017)
Panthera_pardus	Felidae	30.5	51.5	68.852	Carnivory	Solitary	(Wilson *et al.*, 2009; Johnson *et al.*, 2017)
Panthera_tigris	Felidae	121	260	114.88	Carnivory	Solitary	(Wilson *et al.*, 2009; Johnson *et al.*, 2017)
Panthera_uncia	Felidae	35.7	44.8	25.49	Carnivory	Solitary	(Wilson *et al.*, 2009; Johnson *et al.*, 2017)
Pardofelis_temminckii	Felidae	7.9	13.5	70.886	Carnivory	Solitary	(Wilson *et al.*, 2009; Johnson *et al.*, 2017)
Prionailurus_bengalensis	Felidae	2.3	2.9	26.087	Carnivory	Solitary	(Wilson *et al.*, 2009; Johnson *et al.*, 2017)
Prionailurus_planiceps	Felidae	1.7	1.9	11.765	Carnivory	Solitary	(Wilson *et al.*, 2009; Johnson *et al.*, 2017)
Prionailurus_rubiginosus	Felidae	0.9	0.9	0	Carnivory	Solitary	(Wilson *et al.*, 2009; Johnson *et al.*, 2017)
Prionailurus_viverrinus	Felidae	7.5	11.6	54.667	Carnivory	Solitary	(Wilson *et al.*, 2009; Johnson *et al.*, 2017)
Puma_concolor	Felidae	30.7	58.9	91.857	Carnivory	Solitary	(Wilson *et al.*, 2009; Johnson *et al.*, 2017)
Puma_yagouaroundi	Felidae	4.1	5.7	39.024	Carnivory	Solitary	(Wilson *et al.*, 2009; Johnson *et al.*, 2017)
Atilax_paludinosus	Herpestidae	3.25	3.45	6.154	Omnivory	Solitary	(Wilson et al., 2009)
Bdeogale_nigripes	Herpestidae	3.4	3.4	0	Insectivory	Solitary	(Wilson et al., 2009)
Cynictis_penicillata	Herpestidae	0.808	0.808	0	Insectivory	Solitary	(Wilson et al., 2009)
Galerella_pulverulenta	Herpestidae	0.695	0.965	38.849	Carnivory	Solitary	(Wilson et al., 2009)

Galerella_sanguinea	Herpestidae	0.421	0.576	36.817	Carnivory	Solitary	(Wilson et al., 2009)
Helogale_parvula	Herpestidae	0.277	0.282	1.805	Insectivory	Group	(Wilson et al., 2009)
Herpestes_brachyurus	Herpestidae	1.85	2	8.108	Omnivory	Solitary	(Wilson et al., 2009)
Herpestes_ichneumon	Herpestidae	3.15	3.35	6.349	Omnivory	Solitary	(Wilson et al., 2009)
Herpestes_naso	Herpestidae	1.35	3.2	137.04	Omnivory	Solitary	(Wilson et al., 2009)
Herpestes_vitticollis	Herpestidae	2.7	3.4	25.926	Omnivory	Solitary	(Wilson et al., 2009)
Ichneumia_albicauda	Herpestidae	4.05	4.05	0	Insectivory	Solitary	(Wilson et al., 2009)
Mungos_gambianus	Herpestidae	1.6	1.6	0	Insectivory	Group	(Wilson et al., 2009)
Mungos_mungo	Herpestidae	1.365	1.385	1.465	Insectivory	Group	(Wilson et al., 2009)
Suricata_suricatta	Herpestidae	0.484	0.712	47.107	Insectivory	Group	(Wilson et al., 2009)
Crocuta_crocuta	Hyaenidae	59.386	53.665	-9.634	Carnivory	Group	(Wilson *et al.*, 2009; Swanson *et al.*, 2013)
Hyaena_hyaena	Hyaenidae	30	33.5	11.667	Carnivory	Group	(Wilson et al., 2009)
Proteles_cristatus	Hyaenidae	10	10	0	Insectivory	Pair	(Wilson et al., 2009)
Conepatus_humboldtii	Mephitidae	1.53	2.24	46.405	Omnivory	Solitary	(Wilson *et al.*, 2009; Noonan *et al.*, 2016)
Conepatus_mesoleucus	Mephitidae	2.7	3	11.111	Insectivory	Solitary	(Wilson *et al.*, 2009; Noonan *et al.*, 2016)
Mephitis_macroura	Mephitidae	0.72	0.81	12.5	Omnivory	Solitary	(Wilson *et al.*, 2009; Noonan *et al.*, 2016)
Mephitis_mephitis	Mephitidae	2.05	2.6	26.829	Insectivory	Solitary	(Wilson *et al.*, 2009; Noonan *et al.*, 2016)

Spilogale_gracilis	Mephitidae	0.43	0.56	30.233	Omnivory	Solitary	(Wilson *et al.*, 2009; Noonan *et al.*, 2016)
Spilogale_putorius	Mephitidae	0.28	0.4	42.857	Omnivory	Solitary	(Wilson *et al.*, 2009; Noonan *et al.*, 2016)
Spilogale_pygmaea	Mephitidae	0.22	0.24	9.091	Omnivory	Solitary	(Wilson *et al.*, 2009; Noonan *et al.*, 2016)
Aonyx_capensis	Mustelidae	12.2	14.3	17.213	Omnivory	Solitary	(Wilson *et al.*, 2009; Noonan *et al.*, 2016)
Aonyx_cinerea	Mustelidae	3.7	2.5	-32.432	Piscivory	Group	(Wilson *et al.*, 2009; Noonan *et al.*, 2016)
Eira_barbara	Mustelidae	4.4	4.84	10	Omnivory	Solitary	(Wilson *et al.*, 2009; Noonan *et al.*, 2016)
Enhydra_lutris	Mustelidae	24	32	33.333	Piscivory	Solitary	(Wilson *et al.*, 2009; Noonan *et al.*, 2016)
Galictis_cuja	Mustelidae	1	1	0	Carnivory	Variable	(Wilson *et al.*, 2009; Noonan *et al.*, 2016)
Galictis_vittata	Mustelidae	1.8	3.38	87.778	Carnivory	Variable	(Wilson *et al.*, 2009; Noonan *et al.*, 2016)
Gulo_gulo	Mustelidae	10	14.6	46	Carnivory	Solitary	(Wilson *et al.*, 2009; Noonan *et al.*, 2016)
Hydrictis_maculicollis	Mustelidae	4.3	5.75	33.721	Piscivory	Variable	(Wilson *et al.*, 2009; Noonan *et al.*, 2016)
Ictonyx_libyca	Mustelidae	0.39	0.42	7.792	Carnivory	Solitary	(Wilson *et al.*, 2009; Noonan *et al.*, 2016)
Ictonyx_striatus	Mustelidae	0.64	0.91	42.188	Carnivory	Solitary	(Wilson *et al.*, 2009; Noonan *et al.*, 2016)
Lontra_canadensis	Mustelidae	7.9	8.8	11.392	Piscivory	Variable	(Wilson *et al.*, 2009; Noonan *et al.*, 2016)
Lontra_felina	Mustelidae	4	4	0	Piscivory	Solitary	(Wilson *et al.*, 2009; Noonan *et al.*, 2016)
Lontra_longicaudis	Mustelidae	7.9	8.6	8.861	Piscivory	Solitary	(Wilson *et al.*, 2009; Noonan *et al.*, 2016)

Lontra_provocax	Mustelidae	9.4	12.5	32.979	Piscivory	Solitary	(Wilson *et al.*, 2009; Noonan *et al.*, 2016)
Lutra_lutra	Mustelidae	7	10	42.857	Piscivory	Variable	(Wilson *et al.*, 2009; Noonan *et al.*, 2016)
Lutrogale_perspicillata	Mustelidae	7.3	10.29	40.959	Piscivory	Pair	(Wilson *et al.*, 2009; Noonan *et al.*, 2016)
Martes_americana	Mustelidae	0.55	0.8	45.455	Carnivory	Solitary	(Wilson *et al.*, 2009; Noonan *et al.*, 2016)
Martes_flavigula	Mustelidae	2.36	2.83	19.915	Carnivory	Variable	(Wilson *et al.*, 2009; Noonan *et al.*, 2016)
Martes_foina	Mustelidae	1.28	1.72	34.375	Omnivory	Solitary	(Wilson *et al.*, 2009; Noonan *et al.*, 2016)
Martes_martes	Mustelidae	1.34	1.92	43.284	Carnivory	Solitary	(Wilson *et al.*, 2009; Noonan *et al.*, 2016)
Martes_melampus	Mustelidae	1.01	1.56	54.455	Carnivory	Solitary	(Wilson *et al.*, 2009; Noonan *et al.*, 2016)
Martes_pennanti	Mustelidae	2.25	4.5	100	Carnivory	Solitary	(Wilson *et al.*, 2009; Noonan *et al.*, 2016)
Martes_zibellina	Mustelidae	0.9	1.3	44.444	Omnivory	Solitary	(Wilson *et al.*, 2009; Noonan *et al.*, 2016)
Meles_anakuma	Mustelidae	5.4	7.7	42.593	Omnivory	Variable	(Wilson *et al.*, 2009; Noonan *et al.*, 2016)
Meles_leucurus	Mustelidae	6.3	7.1	12.698	Omnivory	Variable	(Wilson *et al.*, 2009; Noonan *et al.*, 2016)
Meles_meles	Mustelidae	8.4	9.2	9.524	Omnivory	Variable	(Wilson *et al.*, 2009; Noonan *et al.*, 2016)
Mellivora_capensis	Mustelidae	6.2	9.4	51.613	Omnivory	Solitary	(Wilson *et al.*, 2009; Noonan *et al.*, 2016)
Mustela_altaica	Mustelidae	0.129	0.236	82.946	Carnivory	Solitary	(Wilson *et al.*, 2009; Noonan *et al.*, 2016)
Mustela_erminea	Mustelidae	0.185	0.32	72.973	Carnivory	Solitary	(Wilson *et al.*, 2009; Noonan *et al.*, 2016)

Mustela_eversmannii	Mustelidae	1.35	2.05	51.852	Carnivory	Solitary	(Wilson *et al.*, 2009; Noonan *et al.*, 2016)
Mustela_frenata	Mustelidae	0.112	0.225	100.89	Carnivory	Solitary	(Wilson *et al.*, 2009; Noonan *et al.*, 2016)
Mustela_itatsi	Mustelidae	0.16	0.49	210.83	Carnivory	Solitary	(Wilson *et al.*, 2009; Noonan *et al.*, 2016)
Mustela_lutreola	Mustelidae	0.61	0.92	52.727	Carnivory	Solitary	(Wilson *et al.*, 2009; Noonan *et al.*, 2016)
Mustela_nigripes	Mustelidae	0.7	1.03	47.143	Carnivory	Solitary	(Wilson *et al.*, 2009; Noonan *et al.*, 2016)
Mustela_nivalis	Mustelidae	0.05	0.09	80	Carnivory	Solitary	(Wilson *et al.*, 2009; Noonan *et al.*, 2016)
Mustela_putorius	Mustelidae	0.71	1.23	73.239	Carnivory	Solitary	(Wilson *et al.*, 2009; Noonan *et al.*, 2016)
Mustela_sibirica	Mustelidae	0.38	0.73	93.6	Carnivory	Solitary	(Wilson *et al.*, 2009; Noonan *et al.*, 2016)
Mustela_subpalmata	Mustelidae	0.21	0.39	86.603	Carnivory	Solitary	(Wilson *et al.*, 2009; Noonan *et al.*, 2016)
Neovison_vison	Mustelidae	0.63	1.16	84.127	Carnivory	Solitary	(Wilson *et al.*, 2009; Noonan *et al.*, 2016)
Poecilogale_albinucha	Mustelidae	0.23	0.32	39.13	Carnivory	Solitary	(Wilson *et al.*, 2009; Noonan *et al.*, 2016)
Pteronura_brasiliensis	Mustelidae	24.5	27.5	12.245	Piscivory	Group	(Wilson *et al.*, 2009; Noonan *et al.*, 2016)
Taxidea_taxus	Mustelidae	6.6	8.2	24.242	Carnivory	Solitary	(Wilson *et al.*, 2009; Noonan *et al.*, 2016)
Vormela_peregusna	Mustelidae	0.38	0.5	32.8	Carnivory	Solitary	(Wilson *et al.*, 2009; Noonan *et al.*, 2016)
Bassaricyon_gabbii	Procyonidae	1.1	1.05	-4.545	Herbivory	Solitary	(Wilson *et al.*, 2009; Noonan *et al.*, 2016)
Bassariscus_astutus	Procyonidae	0.87	1.03	18.391	Omnivory	Solitary	(Wilson *et al.*, 2009; Noonan *et al.*, 2016)

Nasua_narica	Procyonidae	3.95	5.3	34.177	Omnivory	Group	(Wilson *et al.*, 2009; Noonan *et al.*, 2016)
Nasua_nasua	Procyonidae	3.58	4.45	24.302	Omnivory	Variable	(Wilson *et al.*, 2009; Noonan *et al.*, 2016)
Potos_flavus	Procyonidae	2.6	4.4	69.231	Herbivory	Group	(Wilson *et al.*, 2009; Noonan *et al.*, 2016)
Procyon_lotor	Procyonidae	5.94	6.76	13.805	Omnivory	Variable	(Wilson *et al.*, 2009; Noonan *et al.*, 2016)
Procyon_pygmaeus	Procyonidae	3.22	3.7	14.907	Omnivory	Solitary	(Wilson *et al.*, 2009; Noonan *et al.*, 2016)
Ailuropoda_melanoleuca	Ursidae	85	105	23.529	Herbivory	Solitary	(Wilson *et al.*, 2009)
Helarctos_malayanus	Ursidae	56	64	14.286	Omnivory	Solitary	(Wilson *et al.*, 2009)
Melursus_ursinus	Ursidae	72.5	107.5	48.276	Omnivory	Solitary	(Wilson *et al.*, 2009)
Tremarctos_ornatus	Ursidae	70	137.5	96.429	Omnivory	Solitary	(Wilson *et al.*, 2009)
Ursus_americanus	Ursidae	95	142.5	50	Omnivory	Solitary	(Wilson *et al.*, 2009)
Ursus_arctos	Ursidae	165	340	106.06	Omnivory	Solitary	(Wilson *et al.*, 2009)
Ursus_maritimus	Ursidae	200	475	137.5	Carnivory	Solitary	(Wilson *et al.*, 2009)
Ursus_thibetanus	Ursidae	87.5	130	48.571	Omnivory	Solitary	(Wilson *et al.*, 2009)
Genetta_felina	Viverridae	1.7	1.95	14.706	Omnivory	Solitary	(Wilson *et al.*, 2009)
Genetta_genetta	Viverridae	1.85	2.1	13.514	Carnivory	Solitary	(Wilson *et al.*, 2009)
Genetta_johnstoni	Viverridae	2.4	2.4	0	Insectivory	Solitary	(Wilson *et al.*, 2009)
Genetta_maculata	Viverridae	1.9	2.3	21.053	Carnivory	Solitary	(Wilson *et al.*, 2009)

Genetta_pardina	Viverridae	3.1	3.1	0	Omnivory	Solitary	(Wilson *et al.*, 2009)
Genetta_thierryi	Viverridae	1.4	1.4	0	Omnivory	Solitary	(Wilson *et al.*, 2009)
Genetta_tigrina	Viverridae	1.65	1.85	12.12 1	Omnivory	Solitary	(Wilson *et al.*, 2009)
Genetta_victoriae	Viverridae	3	3	0	Omnivory	Solitary	(Wilson *et al.*, 2009)
Poiana_richardsonii	Viverridae	0.455	0.63	38.46 2	Omnivory	Solitary	(Wilson *et al.*, 2009)

References

Johnson, P.J., Noonan, M.J., Kitchener, A.C., Harrington, L.A., Newman, C. & Macdonald, D.W. 2017. Rensching cats and dogs: feeding ecology and fecundity trends explain variation in the allometry of sexual size dimorphism. *Royal Society Open Science* **4**: 170453–7.

Noonan, M.J., Johnson, P.J., Kitchener, A.C., Harrington, L.A., Newman, C. & Macdonald, D.W. 2016. Sexual size dimorphism in musteloids: An anomalous allometric pattern is explained by feeding ecology. *Ecol Evol* 1–7.

Swanson, E.M., McElhinny, T.L., Dworkin, I., Weldele, M.L., Glickman, S.E. & Holekamp, K.E. 2013. Ontogeny of sexual size dimorphism in the spotted hyena (Crocuta crocuta). *J Mammal* **94**: 1298–1310.

Wilson, D.E., Mittermeier, R.A. & Mittermeier, R.A. 2009. *Handbook of the Mammals of the World*. Lynx Edicions.
